# Au_24_Cd Nanoenzyme Coating for Enhancing Electrochemical Sensing Performance of Metal Wire Microelectrodes

**DOI:** 10.3390/bios14070328

**Published:** 2024-07-02

**Authors:** Jia-Yi Chen, Shuang Huang, Shuang-Jie Liu, Zheng-Jie Liu, Xing-Yuan Xu, Meng-Yi He, Chuan-Jie Yao, Tao Zhang, Han-Qi Yang, Xin-Shuo Huang, Jing Liu, Xiao-Dong Zhang, Xi Xie, Hui-Jiuan Chen

**Affiliations:** 1 State Key Laboratory of Optoelectronic Materials and Technologies, School of Electronics and Information Technology, Guangdong Province Key Laboratory of Display Material and Technology, Sun Yat-Sen University, Guangzhou 510006, China; chenjy857@mail2.sysu.edu.cn (J.-Y.C.); liuzhj68@mail2.sysu.edu.cn (Z.-J.L.); xuxy268@mail2.sysu.edu.cn (X.-Y.X.); hemy33@mail2.sysu.edu.cn (M.-Y.H.); yaochj@mail2.sysu.edu.cn (C.-J.Y.); zhangt293@mail2.sysu.edu.cn (T.Z.); yanghq53@mail2.sysu.edu.cn (H.-Q.Y.); huangxsh35@mail.sysu.edu.cn (X.-S.H.); 2 Guangdong Provincial Key Laboratory of Sensor Technology and Biomedical Instrument, School of Biomedical Engineering, Sun Yat-Sen University, Shenzhen 518107, China; huangsh239@mail.sysu.edu.cn; 3 Tianjin Key Laboratory of Brain Science and Neural Engineering, Academy of Medical Engineering and Translational Medicine, Tianjin University, Tianjin 300072, China; liushuangjie@tju.edu.cn (S.-J.L.); xiaodongzhang@tju.edu.cn (X.-D.Z.); 4 The First Affiliated Hospital of Sun Yat-Sen University, Guangzhou 510080, China; liuj753@mail.sysu.edu.cn

**Keywords:** electrochemical, nanoenzymes, neurotransmitter, differential pulse voltammetry, microelectrode

## Abstract

Dopamine (DA), ascorbic acid (AA), and uric acid (UA) are crucial neurochemicals, and their abnormal levels are involved in various neurological disorders. While electrodes for their detection have been developed, achieving the sensitivity required for in vivo applications remains a challenge. In this study, we proposed a synthetic Au_24_Cd nanoenzyme (ACNE) that significantly enhanced the electrochemical performance of metal electrodes. ACNE-modified electrodes demonstrated a remarkable 10-fold reduction in impedance compared to silver microelectrodes. Furthermore, we validated their excellent electrocatalytic activity and sensitivity using five electrochemical detection methods, including cyclic voltammetry, differential pulse voltammetry, square-wave pulse voltammetry, normal pulse voltammetry, and linear scanning voltammetry. Importantly, the stability of gold microelectrodes (Au MEs) modified with ACNEs was significantly improved, exhibiting a 30-fold enhancement compared to Au MEs. This improved performance suggests that ACNE functionalization holds great promise for developing micro-biosensors with enhanced sensitivity and stability for detecting small molecules.

## 1. Introduction

Dopamine (DA), ascorbic acid (AA), and uric acid (UA) are three important small-molecule compounds present in physiological fluids, which are of great research interest because of their irreplaceable roles in the regulation of central nervous activity or blood vessel properties [1,2,3]. One of these, DA, is an excitatory neurotransmitter that facilitates the transmission of information from the brain, so abnormal levels of DA can lead to psychiatric disorders in the body [4]. For example, underproduction of DA can lead to diseases such as Alzheimer’s and Parkinson’s, and overproduction of DA can lead to symptoms such as extreme hyperactivity and inability to control emotions [5,6]. AA, also known as vitamin C, is a soluble vitamin. AA can promote the repair of metabolic tissues by participating in processes such as remodeling chromatin structures, or it has antioxidant functions by inhibiting the destruction of free radicals [7,8]. Disturbed AA secretion levels can lead to neurological disorders and even cancer and other related diseases. UA is a major product of purine metabolism, and diseases such as cerebrovascular disease and Alzheimer’s disease are associated with its abnormal secretion [9,10]. DA, AA, and UA are three neurotransmitters found in low concentrations in the extracellular fluid of human brain tissue. DA is usually in the submicromolar range, and AA and UA are usually in the millimolar range [11,12].

Current research is mainly focused on the development of electrodes with fast responses to detect this class of small-molecule compounds, such as glassy carbon electrodes, bulk Au or Pt electrodes, etc. [4,10,13,14]. In addition, a large amount of research has also been conducted on sensors that can quickly and selectively respond and cost effectively detect precursor substances of neurotransmitters such as DA [15,16]. Various molecular techniques, such as liquid chromatography (LC), mass spectrometry (MS), and capillary electrophoresis (CE), have been used for the detection of DA, UA, and AA [17]. However, LC and MS have the disadvantage of expensive instrumentation that is difficult to popularize, and CE has the disadvantage of underperformance in production quality [18,19,20]. In most methods, electrochemical methods have the advantages of low cost, simplicity of operation, rapid response to biologically active molecules, and high sensitivity [11,17,21]. During electrochemical detection, the target analyte undergoes a redox reaction on the surface of the electrode, and the transfer of electrons constitutes the signal of electrochemical detection [22,23]. Based on electrochemical methods of detection, the oxidation process of DA consists of three main steps: the formation of o-dopamine quinone after the exchange of two electrons and two protons, further intramolecular cyclization to produce white epileptic polyamino pigments, and finally, the oxidation of dopamine pigments [24]. The oxidation of UA is also widely believed to proceed via a 2e^−^, 2H mechanism, generating an unstable diimide [25]. AA is oxidized on the surface of the electrodes as dehydroascorbic acid, thereby releasing two electrons [26]. Abellán-Llobregat et al. reported on a graphene oxide electrochemical sensor based on gold nanoparticles for simple and rapid quantification of AA and UA in biofluids by chronoamperometry, with excellent results [27]. Zülfikar Temoc¸ et al. reported on a modified glassy carbon electrode with high sensitivity for electrochemical detection [28]. In electrochemical techniques, cyclic voltammetry (CV), differential pulse voltammetry (DPV), square-wave voltammetry (SWV), conventional pulse voltammetry (NPV), and linear scanning voltammetry (LSV) are widely used in several fields. In the CV technique, the potential is linear with respect to time, and the redox behavior can be rapidly observed over a wide range of potentials. LSV has attracted great interest because of its advantages of low cost, fast response, and simplicity. The pulsed techniques, such as DPV, SWV, etc., all have good sensitivity, accuracy, and signal-to-noise ratios. The advantage of SWV is a faster scanning rate, which enables the analysis time to be greatly reduced. NPV is a pulsed detection technique that maintains the electrode at a constant base potential by applying a series of linearly increasing pulse potentials to the working electrode, which has the advantage of a fast updating rate and allows for a fast and good response under certain circumstances [29,30,31,32]. Therefore, it is critical to select the appropriate electrochemical technique to achieve highly sensitive detection of analytes.

Nanoparticles are usually able to enhance the specific surface area of an electrode to improve electrochemical properties such as electrode sensitivity [33,34,35]. For example, Lili Feng et al. reported on a nanostructure of Au/Pt nanodot shells grown on gold nanorods, which had superior catalytic activity due to its high specific surface area and volume [34], and Yaoguang Wang et al. reported that a glassy carbon electrode modified with gold nanoparticles and thiol graphene showed excellent performance in detecting Hg [35]. Typically, catalysts are able to oxidize the target analytes at high reaction rates and low overpotentials, thereby increasing the sensitivity of the electrodes [33,36]. Therefore, the development of catalytic electrodes capable of detecting DA, AA, and UA with high sensitivity is essential for improving electrode characteristics [37]. Natural enzymes, which are catalysts involved in most biological life reactions, are highly specific and catalytic for reaction substrates. Modification of conventional electrodes using natural enzymes can greatly improve the sensing sensitivity of specific electrodes. However, due to the disadvantages of difficulty in collection, high cost, and susceptibility to denaturation by environmental influences, researchers have been searching for a promising artificial enzyme to replace the natural ones [38,39,40]. In the past years, some nanoenzymatic materials with superior performance have been reported, including platinum nanoparticles [41,42], carbon nanotubes [43,44], and gold nanoparticles [45,46,47,48]. Nanoenzymes have been studied as highly active enzyme-like catalysts due to their high stability, low cost, and tunable catalytic activity.

**Figure 1:**
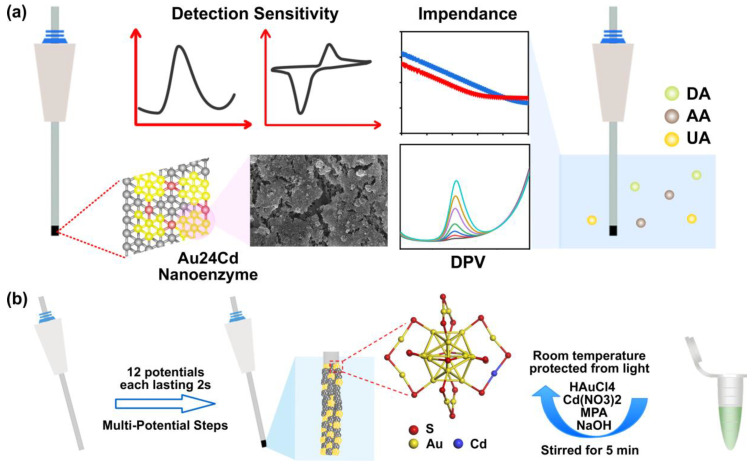
ACNE microelectrode detection process and preparation method. (**a**) Illustration of ACNE microelectrode structure and sensing performance. The metal microelectrode-sensing portion was uniformly distributed with nanoenzyme clusters in about a 1 mm area. The prepared ACNE microelectrodes were tested in DA, AA, and UA solutions by electrochemical methods such as DPV, CV, and EIS, which were used to examine the sensitivity and sensing properties of the electrodes. (**b**) ACNE microelectrode preparation method. ACNEs were prepared first, and the reaction was carried out at room temperature in an environment protected from light. HAuCl_4_ aqueous solution, Cd (NO_3_)_2_ aqueous solution, and NaOH aqueous solution were added into the same container at the desired concentrations and stirred constantly during the process to fully react the solutions to produce ACNEs, and then the ACNEs were uniformly deposited on the metal microelectrodes by multi-potential steps in the 1 mm long portion of the microelectrode-sensing area.

In this study, we report on electrochemical microelectrodes based on Au_24_Cd nanoenzymes (ACNEs) for the highly sensitive detection of DA, AA, and UA, as shown in Figure 1. The modification of ACNEs onto the surface of the metal electrodes resulted in the presence of a sufficient number of active sites on the surface, which enhanced the rate of electron transfer during the catalytic reaction. To investigate the effect of ACNEs on the sensing efficiency of different metal electrodes, we used electrochemical impedance spectroscopy (EIS) and CV to characterize gold microelectrodes (Au MEs), silver microelectrodes (Ag MEs), Au MEs with ACNE modification (ACNE/Au MEs), and Ag MEs with ACNE modification (ACNE/Ag MEs). The results showed that ACNE modification reduced the low-frequency impedance of the metal electrodes. Moreover, the results showed that the repeatability of the gold-based electrodes was superior to that of the silver-based electrodes. Secondly, to investigate the sensing efficiency of ACNE microelectrodes in different electrochemical detection methods, we explored the detection performance of ACNE/Au MEs for DA, UA, and AA by four methods, including DPV, SWV, NPV, and LSV. The comparative results illustrated that the ACNEs possessed the effect of decreasing the electrode overpotential and increasing the detection sensitivity in different detection methods. Therefore, ACNE/Au MEs have potential for the development of a highly sensitive response to changes in small-molecule compounds such as DA, AA, and UA at low overpotentials and low concentrations, making it beneficial for them to be further developed as smart biosensors for biomedical applications. 

## 2. Materials and Methods

### 2.1. Preparation of ACNEs

In this work, nanoenzyme clusters that selected thiolate that had opposite charges as ligands were used, and the reaction was carried out at room temperature and under a light-protection setup. Specifically, HAuCl_4_ aqueous solution (20 mM, 0.25 mL), Cd(NO_3_)_2_ aqueous solution (20 mM, 10 μL), and MPA aqueous solution (5 mM, 2 mL) were added to ultrapure water (2.35 mL) and stirred continuously for 5 min. During stirring, Au atoms in the HAuCl_4_ were replaced by Cd^2+^ in Cd(NO_3_)_2_ at a molar ratio of 24:1 to synthesize Au_24_Cd nanoenzyme clusters. The aqueous NaOH solution (1 M, 0.3 mL) was continuously added dropwise to the pH = 12 of the reaction solution, and then 0.1 mL of NaBH_4_ solution was added. At this point, the NaOH solution (10 mL, 0.2 M) reacted and fused with 43 mg of NaBH_4_ powder, so the solution became clear and transparent. Next, saturated CO_2_ was passed into the confinement for 2 min. after stirring for 3 h, and the brown solution was lyophilized to obtain the pure Au_24_Cd product.

### 2.2. Preparation of ACNE Microelectrodes

For the Ag wire-based ACNE microelectrode, a sliver microelectrode with a diameter of 0.15 mm was sandpapered and then placed in distilled water and sonicated for 10 min to remove surface impurities. Then, 0.5 g of Au_24_Cd powder was taken and mixed thoroughly with 10 mL of deionized water to obtain 5 mg/mL of nanoenzyme solution. A three-electrode electrochemical system using a commercial Ag/AgCl electrode as the reference electrode, a platinum sheet electrode as the counter electrode, and a sliver microelectrode as the working electrode was used for the deposition of the nanoenzymes by multi-potential steps (STEP) in a CHI760E electrochemical workstation. Immediately after that, the electrodeposition parameters were set up with 12 potentials at voltages ranging from 1.7–3.4 V, with a step amplitude of 0.2 V between the potentials and a duration of each potential of 2 s. To ensure the uniformity of the nanoenzymes deposited on the surface of the sliver microelectrode, the deposition current was controlled to be less than 0.4 mA, and then electrodeposition was started. Finally, the as-prepared nanoenzyme electrode was insulated with polyimide (PI), while the ~1 mm electrode tip was retained uninsulated in order to be used as a sensing area. The ACNE/silver microelectrode was obtained after the PI insulating layer was cured at room temperature for 12 h. For the Au-wire-based nanoenzyme electrodes, Au MEs with a diameter of 0.1 mm were polished and then placed in distilled water and sonicated for 10 min to remove surface impurities. Similarly, the Au MEs were subjected to nanoenzyme deposition using an electrochemical three-electrode system.

### 2.3. DA, AA, and UA Solution Preparation

PBS (Biosharp, Guangzhou, China) was used as the solvent for the DA, UA, and AA solutions, and the pH of the PBS was 7.2. The DA, UA, and AA in this experiment were purchased from Aladdin. In detail, for the DA solution, 0.984 g of DA powder was taken and fully dissolved in 10 mL of PBS solution to obtain a 50 mM DA solution. Then 2 mL, 50 mM of DA solution was taken in 8 mL of PBS solution and fully mixed to obtain a 10 mM DA solution. Similarly, 1 mL, 10 mM of DA solution was placed in 9 mL of PBS solution to obtain 1 mM of DA solution. Finally, 1 mL, 1 mM of DA solution was dissolved in 9 mL of PBS solution to obtain 100 nM of DA. For the UA solution, 0.0067 g of UA powder was taken and fully dissolved in 10 mL of PBS solution to obtain 40 mM of UA solution, which was used in subsequent drop test experiments. For AA solution preparation, 0.1761 g of AA powder was weighed and fully dissolved in 10 mL of PBS solution to obtain a 10 mM AA solution, which was used in the subsequent drop test. Considering the chemical instability of the above solutions, all of the above solutions needed to be prepared and used immediately and stored at room temperature and protected from light.

### 2.4. Surface Morphological Characterization and Elemental Analysis of Electrodes

In this study, the surface morphology of the Au MEs and Ag MEs before and after modification of the nanoenzymes was observed by scanning electron microscopy (SEM, Thermo Fisher Scientific Apreo (2S+)). In our previous work, we have already characterized the basic features of the nanoenzyme electrodes.

### 2.5. Electrochemical Characterization of Electrodes

All of the following electrochemical tests were performed at room temperature utilizing a CHI760E electrochemical workstation. The experiment was performed using a three-electrode system for electrochemical characterization, with Ag/AgCl as the reference electrode, a platinum electrode as the counter electrode, and the test electrodes (Ag ME, ACNE/Ag ME, Au ME, and ACNE/Au ME) as the working electrodes. During the test, we varied the solution concentration by changing the test solution, pausing the test when changing the solution, and leaving it for 30 s to stabilize the solution before continuing the test.

When testing DA solutions with different concentrations by the DPV, SWV, NPV, and LSV methods, DA solutions in the concentration ranges of 0.1–10 μM and 30–300 μM were tested. At the low concentration range of DA solutions (0.1–10 μM), the DA solutions were made to vary in the concentration range of 0.1–10 μM by adding 100 nM or 1 μM of DA of solution dropwise to 10 mL of PBS solution. At the high concentration range of DA solutions (30–300 μM), the DA solutions were made to vary in the concentration range of 30–300 μM by adding 10 μM of DA solution. When detecting DA with different electrochemical methods, the parameters were set as follows: For DPV, we set the scanning voltage of Init to −0.2 V, Final to 0.6 V, Incr to 0.004 V, Amplitude to 0.05 V, Pulse Width to 0.05 s, Sampling Width to 0.02 s, and Pulse Period to 0.5 s. For SWV, we set the parameters of Init to −0.2 V, Final to 0.6 V, Incr to 0.004 V, Amplitude to 0.025 V, and Frequency to 5 Hz. For NPV, we set the parameters of Init to −0.2 V, Final to 0.6 V, Incr to 0.004 V, Sampling Width to 0.02 s, and Pulse Period to 0.2 s. For LSV, we set the parameters of Init to −0.2 V, Final to 0.6 V, Scan Rate to 0.1 V/s, and Sample Interval to 0.001 V.

Similarly, UA solutions with concentrations of 100–600 μM were tested with DPV, SWV. When detecting UA with DPV and SWV, the parameters were set as follows: in DPV, we set the scanning voltage of Init to −0.1 V, Final to 0.7 V, Incr to 0.004 V, Amplitude to 0.05 V, Pulse Width to 0.05 s, Sampling Width to 0.02 s, and Pulse Period to 0.5 s. In SWV, we set the parameters of Init to −0.1 V, Final to 0.7 V, Incr to 0.004 V, Amplitude to 0.025 V, and Frequency to 4 Hz. AA solutions with concentrations of 1000–2000 μM were tested with DPV, SWV. When detecting AA with DPV and SWV, the parameters were set as follows: in DPV, we set the scanning voltage of Init to −0.2 V, Final to 0.6 V, Incr to 0.004 V, Amplitude to 0.05 V, Pulse Width to 0.05 s, Sampling Width to 0.02 s, and Pulse Period to 0.5 s. For SWV, we set the parameters of Init to −0.1 V, Final to 0.7 V, Incr to 0.004 V, Amplitude to 0.025 V, Frequency to 5 Hz.

## 3. Results and Discussion

**Figure 2:**
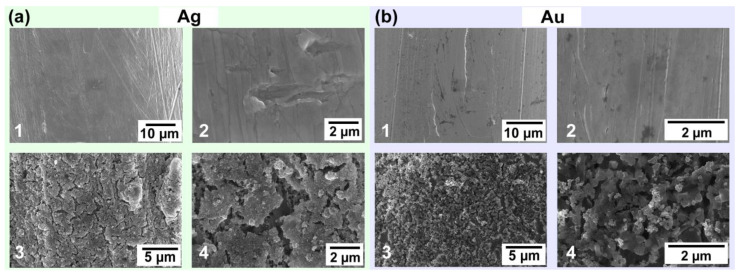
Characterization pictures of ACNE microelectrodes. (**a1**) is a SEM image of a Ag ME at a magnification of 2000×, and (**a2**) is a SEM image of a Ag ME at a magnification of 20,000×. (**a3**) is a SEM image of an ACNE/Ag ME at a magnification of 5000×, and (**a4**) is a SEM image of an ACNE/Ag ME at a magnification of 20,000×. (**b1**) is a SEM image of a Au ME at a magnification of 2500×, and (**b2**) is a SEM image of a Au ME at a magnification of 20,000×. (**b3**) is a SEM image of an ACNE/Au ME at a magnification of 5000×, and (**b4**) is a SEM image of an ACNE/Ag ME at a magnification of 20,000×.

We prepared ACNE microelectrodes to explore their enhancement effect on DA-, AA-, and UA-sensing sensitivity. In order to investigate the effects of the atomic-layer interfaces of the different metals on the performance of nanoenzyme electrodes, we used Au MEs and Ag MEs with excellent electrical properties as carrier electrodes for modification and characterization. For Au MEs, ACNEs were deposited on a 0.1 mm diameter gold wire using the multi-potential step (STEP) method. The ACNE/Au ME was obtained by scanning 10 turns at potentials from 1.7 V to 3.4 V. Then scanning electron microscopy (SEM) was used to characterize the morphology of the ACNE/Au ME. Figure 2(b1) is a SEM image of a Au ME surface magnified 2500 times, and Figure 2(b2) is a SEM image of a Au ME surface magnified 20,000 times. It shows that the Au ME surface was smooth. Figure 2(b3) shows a SEM image of an ACNE/Au ME surface magnified 5000 times, and Figure 2(b4) shows a SEM image of an ACNE/Au ME surface magnified 20,000 times, which shows that the Au ME surface was uniformly distributed, with 20 layers of thick ACNEs. For the Ag ME, ACNEs were electrodeposited by the STEP method on a silver wire with a diameter of 0.15 mm. An ACNE/Ag ME with 500 nm of ACNEs deposited on the surface was also scanned. Similarly, a SEM image of the Ag ME magnified 2000× is shown in Figure 2(a1), and a Ag ME SEM image magnified 20,000×, shown in Figure 2(a2), showed that the surface of the Ag ME was flat and smooth, and the surface of the microelectrode with deposited ACNEs was uniformly distributed with clusters of atoms, as shown in Figure 2(a3,a4). This shows that both the roughness and specific surface area of the electrode surface increased after the deposition of ACNEs. This feature may lead to a decrease in the impedance of the modified electrode. This is mainly due to the involvement of nanoparticles on the surface of the electrode in the reaction and the increase in electron transfer when voltage is applied to the metal microelectrode deposited with ACNEs.

**Figure 3:**
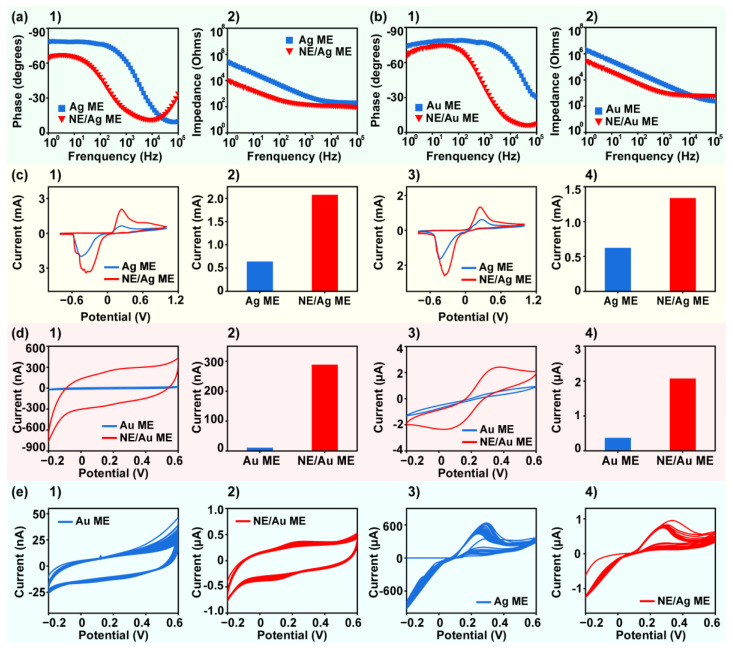
Electrochemical characterizations and stability testing of ACNE microelectrodes. Bode plots of (**a1**) Ag ME and (**a2**) ACNE/Ag ME. The bode plots were obtained by electrochemical impedance detection in PBS. (**b1**,**b2**) Bode plots of Au ME modified with ACNEs and unmodified Au ME, which were obtained by electrochemical impedance testing in PBS. Comparison plots of (**c1**) CV curves and (**c2**) peak oxidation currents of Ag ME modified with ACNEs and unmodified Ag ME when the electrodes were scanned in PBS. Comparison of (**c3**) CV curves and (**c4**) peak oxidation currents of Ag ME modified with ACNEs and unmodified Ag ME when the electrodes were scanned in K_3_[Fe(CN)]_6_. Comparison plots of (**d1**) CV curves and (**d2**) peak currents of Au ME modified with ACNEs and unmodified Au ME when the electrodes were scanned in PBS (at 0.3 V voltage). Comparison plots of (**d3**) CV curves and (**d4**) peak currents of Au ME modified with ACNEs and unmodified Au ME when the electrodes were scanned in K_3_[Fe(CN)]_6_ (at 0.3 V voltage). CV curve plots of (**e1**) Au ME and (**e2**) Au ME modified with ACNEs in PBS solution for 50 repetitive scans; CV curve plots of (**e3**) Ag ME and (**e4**) Ag ME modified with ACNEs in PBS solution for 50 repetitive scans.

To verify that ACNEs can enhance the electrochemical performance of metal electrodes from multiple perspectives, we first examined the coated electrodes by EIS and CV. In specific, an Ag ME, a Au ME, an ACNE/Ag ME, and an ACNE/Au ME were characterized by EIS and CV using an electrochemical workstation. EIS is an effective method for assessing the charge transfer processes associated with electrode surfaces by representing the electron transfer kinetics at the electrode interface at high frequencies through the semicircular diameter [49,50,51]. At a lower frequency, the linear part indicates diffusion processes. Low conductivity indicates weak electron transfer between the electrode and solution interface, which is represented by larger semicircles. We first performed EIS tests on the four types of electrodes with the impedance detection frequency set at 0.1 Hz–10^5^ Hz. Figure 3(a1,a2) show the bode plots of the Ag ME and the ACNE/Ag ME, which demonstrate the frequency-phase and frequency-impedance plots of the electrodes, respectively. Figure 3(a2) shows the comparative frequency-impedance plots of the Ag ME and ACNE/Ag ME. The Ag ME modified with ACNEs had lower impedance, and we compared the impedance values of the two electrodes at 1 Hz. The impedance of the Ag ME modified with ACNEs was 10 times lower than that of the unmodified Ag ME, demonstrating that ACNEs have the characteristic of reducing the impedance of Ag MEs. The results showed that the modification of ACNEs enabled the Ag ME to obtain lower impedance, and the electrochemical performance of the Ag ME was significantly improved. Similarly, we characterized the electrical properties of a Au ME modified with ACNEs and an unmodified Au ME using the EIS test. Figure 3(b1,b2) show a comparison of the bode plots of the Au ME and the ACNE/Au ME, and the impedance of the ACNE/Au ME was six times lower than that of the Au ME when comparing the impedance values at an excitation frequency of 1 Hz. Therefore, for the Ag ME and the Au ME, the low-frequency impedance of the electrodes was reduced more substantially after the modification of ACNEs. The above results demonstrate the potential of ACNEs to enhance the electrochemical performance of the electrodes.

We also characterized the intrinsic electrical properties of the electrodes using the CV method in PBS and 5.0 mM [Fe(CN)_6_]^3−^/[Fe(CN)_6_]^4−^ in 0.1 M KCl solution (K_3_[Fe(CN)]_6_). PBS is considered an ideal salt solution for short-term characterization electrodes in vitro, and K_3_[Fe(CN)]_6_ is a commonly used electrochemical probe. In addition, CV is often considered to be a commonly used characterization technique that allows for rapid analysis of the target analyte over a wide range. The more pronounced current response in the CV curve may be due to the faster rate of electron transfer at the electrode surface [52,53]. When CV was performed in PBS, the CV curve response was higher for the ACNE/Ag ME than the Ag ME, as shown in Figure 3(c1). For a more direct response to the corresponding results of the CV curves of the two electrodes, we compared the oxidation peaks of the Ag ME and the ACNE/Ag ME, as shown in Figure 3(c2). The results showed that the peak oxidation potential of the ACNE/Ag ME in PBS was higher, at about 2 mA, which is three times higher than the 0.6 mA oxidation peak of the Ag ME. The CV test in K_3_[Fe(CN)]_6_ solution also exhibited a higher oxidation peak potential for the ACNE/Ag ME, as shown in Figure 3(c3). And we also compared the oxidation peaks of the Ag ME and the ACNE/Ag ME in K_3_[Fe(CN)]_6_ solution, as shown in Figure 3(c4). Due to the peak profile generated in PBS, the appearance of this peak is believed to be caused by the instability of the silver material under positive voltage, which can affect the specific detection results of subsequent sensing experiments. Therefore, Ag MEs are not considered the best choice for electrochemical sensing experiments. We used the Au ME and the ACNE/Au ME for CV tests in PBS and K_3_[Fe(CN)]_6_ solutions, respectively. Based on the Au ME, there was no oxidation peak potential in PBS, which is shown in Figure 3(d4). This CV result demonstrated the more stable electrical characteristics of the Au ME under positive voltage conditions. Figure 3(d2) compares the Au ME modified with ACNEs, showing a more pronounced peak current at 0.3 V, which is 26 times higher than the peak current value of the Au ME. These results demonstrated that the ACNE/Au ME had higher sensitivity than the Au ME for CV detection in PBS. Similarly, we compared the CV test curves of the Au ME and the ACNE/Au ME in K_3_[Fe(CN)]_6_ solutions, as shown in Figure 3(d3). The Au ME showed a weaker current response, and the ACNE/Au ME exhibited a stronger current response, while the redox peak of the CV curve of the ACNE/Au ME is obvious. Figure 3(d4) more visually compares the current response amplitude of the two electrodes at 0.3 V. The results showed that the peak current of the ACNE/Au ME was about 2 μA, which is five times higher than the 0.37 μA for the Au ME. The above results indicate that the ACNE/Au ME is more electrically active than the Au ME; this may be due to the fact that ACNE/Au MEs have a larger specific surface area, leading to an electron transfer process that appears to be faster and quasi-reversible. In addition, the electrochemical performance of Au MEs is more stable compared with Ag MEs.

To further validate the repeatability of the electrodes, we performed a CV test in PBS with 50 cycle scans for a Au ME, an ACNE/Au ME, a Ag ME, and an ACNE/Ag ME. The experiment was designed to assess the stability of the electrodes in use based on the degree of scan overlap of the curves. It was observed that the CV curves of the Au ME had a poorer degree of coincidence, as shown in Figure 3(e1). The CV curves of the ACNE/Au ME showed a better degree of coincidence, as shown in Figure 3(e2). To compare the overlap of the curves more specifically, we indicated the degree of CV curve overlap for the Au ME by calculating the variance value of 50 currents at 0.3 V. The results showed that the CV curves of the ACNE-modified microelectrodes were better than those of the ACNE/Au ME. The results showed that the current variance value of the ACNE/Au ME was 30 times higher than that of the Au ME. This result illustrated that the ACNE/Au ME had higher repeatability in CV testing than the Au ME. Similarly, the Ag ME and the ACNE/Ag ME were placed in PBS solution for 50 laps of scanning CV tests. Poor overlap of CV curves was observed for both the Ag ME and the ACNE/Ag ME, as shown in Figure 3(e3), and the ACNE/Ag ME, as shown in Figure 3(e4). In summary, modified ACNEs can reduce the electrode impedance so that the sensing electrode presents a more stable detection trend and a more sensitive response current. Due to the instability of Ag material, additional oxidation reactions are prone to interfere with the results of the experimental group under positive pressure conditions, so we utilized the Au ME as the base electrode to further investigate the enhancement effect of the ACNE/Au ME on the detection of small-molecule compounds, such as DA, AA, UA, and so on.

**Figure 4:**
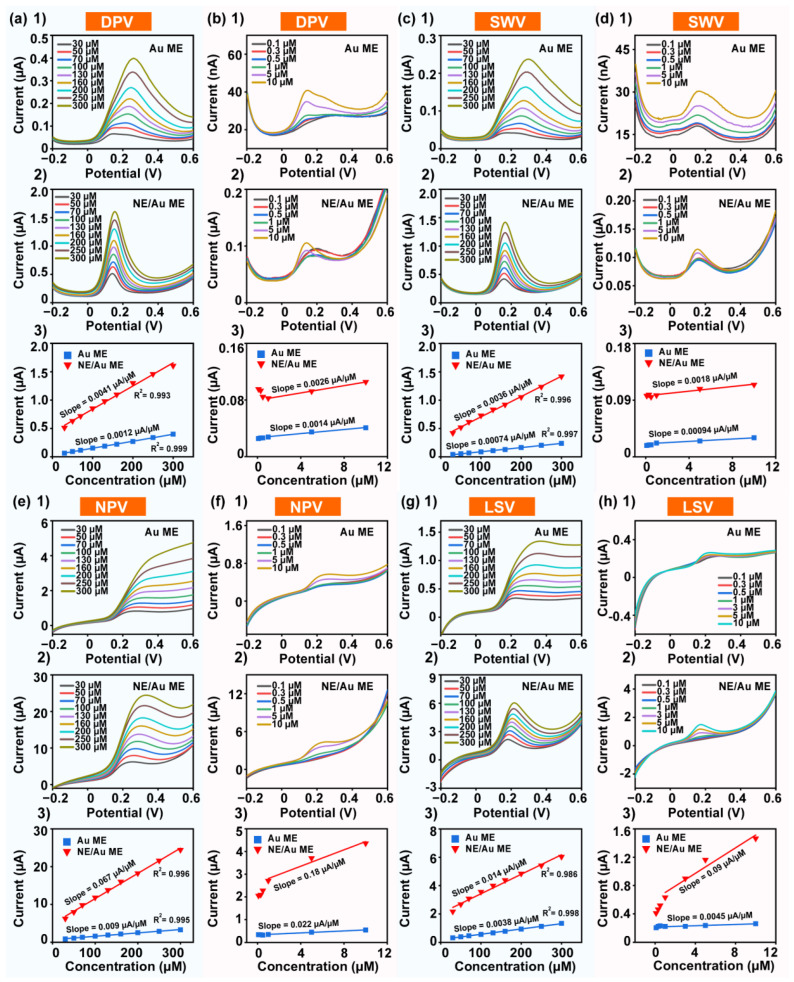
Characterization of electrochemical performance of ACNE/Au ME in high-concentration DA solution (30 μM–300 μM) and low-concentration DA solution (0.1 μM–10 μM). (**a1**) DPV curves of Au microelectrode in high-concentration DA solution; (**a2**) DPV curves of ACNE/Au ME in high-concentration DA solution; (**a3**) peak currents of the relations between concentration and DPV responses of Au ME and ACNE/Au ME in high-concentration DA solution. (**b1**) DPV curve of Au ME in low-concentration DA solution; (**b2**) DPV curve of ACNE/Au ME in low-concentration DA solution; (**b3**) peak currents of the relations between concentration and DPV responses of Au ME and ACNE/Au ME in low-concentration DA solution. (**c1**) SWV curves of Au ME in high-concentration DA solution; (**c2**) SWV curves of ACNE/Au ME in high-concentration DA solution; (**c3**) peak currents of the relations between concentration and SWV responses of Au ME and ACNE/Au ME in high-concentration DA solution. (**d1**) SWV curves of Au ME in low-concentration DA solution; (**d2**) SWV curves of ACNE/Au ME in low-concentration DA solution; (**d3**) peak currents of the relations between concentration and SWV responses of Au ME and ACNE/Au ME in low-concentration DA solution. (**e1**) NPV curve of Au microelectrode in high-concentration DA solution; (**e2**) NPV curve of ACNE/Au ME in high-concentration DA solution; (**e3**) peak currents of the relations between concentration and NPV responses of Au ME and ACNE/Au ME in high-concentration DA solution. (**f1**) NPV curve of Au microelectrode in low-concentration DA solution; (**f2**) NPV curve of ACNE/Au ME in low-concentration DA solution; (**f3**) peak currents of the relations between concentration and NPV responses of Au ME and ACNE/Au ME in low-concentration DA solution; (**g1**) LSV curve of Au ME in high-concentration DA solution; (**g2**) LSV curve of ACNE/Au ME in high-concentration DA solution; (**g3**) peak currents of the relations between concentration and LSV responses of Au ME and ACNE/Au ME in high-concentration DA solution. (**h1**) LSV curve of Au microelectrode in low-concentration DA solution; (**h2**) LSV curve of ACNE/Au ME in low-concentration DA solution; (**h3**) peak currents of the relations between concentration and LSV responses of Au ME and ACNE/Au ME in low-concentration DA solution.

The electrochemical detection performances of the ACNE/Au ME for DA, AA, and ΜA detection were evaluated using four electrochemical methods, including DPV, SWV, NPV, and LSV. Of all electrochemical analysis methods, pulsed techniques are considered more sensitive. DPV is an electrochemical detection pulsing technique that superimposes voltage pulses on a linear scanning potential and rapidly samples the current before each potential change [30]. As a result, DPV reduces the effect of the charging current, resulting in higher sensitivity. First, the DA solutions with different concentration gradients were detected and analyzed using the DPV method. In order to show the detection results of the electrode at different concentration gradients more clearly, we performed solution testing and analysis in a high-concentration DA solution of 30–300 μM and a low-concentration DA solution of 0.1–10 μM. Figure 4(a1) demonstrates the DPV curves of the Au ME for the detection of high-concentration DA solutions, and Figure 4(a2) demonstrates the DPV curves of the ACNE/Au ME for the detection of high-concentration DA solutions. The results showed that the Au ME responded more slowly to DA, detected higher overpotentials for peak currents, exhibited broader peak curves, and had peak response curves that presented as broad and flat at relatively low concentrations. On the other hand, the ACNE/Au ME showed lower and more stable overpotentials and a sharp peak curve when detecting high concentrations of DA solution. A sharp detection peak represents a high reaction rate; therefore, the ACNE/Au ME had a higher reaction rate than the Au ME. Further, to visually compare the sensitivity of the ACNE/Au ME and the Au ME in detecting high concentrations of DA, we fitted the relation between concentrations and DPV responses of the Au ME and the ACNE/Au ME, as shown in Figure 4(a3). The results showed that the slope of the fitted linear equation for the Au microelectrode was 0.0012 μA/μM with R^2^ = 0.999, whereas the slope of the linear equation fitted for the ACNE/Au ME was 0.0041 μA/μM with R^2^ = 0.993. Therefore, these results indicate that ACNE/Au MEs can detect high-concentration DA solutions linearly and sensitively using DPV; meanwhile, the detection sensitivity of the ACNE/Au ME was improved by 3.4-fold compared with that of the Au ME. Next, we tested the effect of ACNE modification on the sensitivity of the Au ME for the detection of lower concentrations of DA solution. Figure 4(b1) demonstrates the DPV curves of the Au ME for the detection of low-concentration DA solutions, and Figure 4(b2) demonstrates the DPV curves of the ACNE/Au ME for the detection of low-concentration DA solutions. The DPV curve of the Au ME at low concentrations of DA solution showed instability, and the peak potential of the reaction was not consistent with that of the DPV curve at high concentrations, while the DPV curves obtained by the ACNE/Au ME at low concentrations of DA solution showed stable and sharp peaks with consistent peak potentials. The slopes of the sensing fitting equations were 0.0012 μA/μM for the Au ME and 0.0026 μA/μM for the ACNE/Au ME, as shown in Figure 4(b3). These results indicated that the ACNE/Au ME could detect the peak potentials of low-concentration DA solutions by the DPV detection method for highly sensitive detection and fast response to low-concentration DA solutions. In summary, the modification of ACNEs can enhance the sensing sensitivity and stability of Au MEs when detecting DA solutions using the DPV method.

SWV can be regarded as a special case of DPV, differing from DPV in that SWV is regarded as a pair of symmetrical square-wave pulses of uniform width and opposite direction superimposed on a linear scanning potential [31]. The first pulse in each cycle collects the forward current, and the second pulse collects the reverse current. As a result, the net current is high and the charging current can be effectively differentiated. In contrast, SWV may possess higher detection sensitivity and a faster response, so we further investigated the effect of ACNEs on the electrochemical detection performance of DA solution by the SWV method. Similarly, we quantitatively tested and analyzed a high-concentration DA solution of 30–300 μM, as shown in Figure 4(c1), and a low-concentration DA solution of 0.1–10 μM, as shown in Figure 4(c2). The results showed that the response oxidation peaks of the SWV curves of the Au microelectrodes in higher-concentration DA solutions were relatively flat, especially at lower concentrations, where the response peaks were not obvious. However, the ACNE/Au ME showed a sharp oxidation reaction peak and stable overpotential value when detecting high-concentration DA solutions using the SWV method. Figure 4(c3) shows the relation between high DA concentrations and the SWV responses of the Au ME and the ACNE/Au ME. The slopes of the linear equations were ascertained to be 0.00074 μA/μM with R^2^ = 0.997 for the Au ME and 0.0036 μA/μM with R^2^ = 0.996 for the ACNE/Au ME. These results demonstrated that the modification of ACNEs resulted in an approximately 5-fold increase in the sensing sensitivity of the Au ME to DA solutions using the SWV method of detection. In addition, SWV detection of low-concentration DA solutions was performed with the Au ME and the ACNE/Au ME. Comparing the results of the Au ME and ACNE/Au ME assays, Figure 4(d4) shows that the SWV curves of the Au ME test exhibit an unstable and drifting baseline, whereas the SWV curves of the ACNE/Au ME test exhibit a stable baseline with consistent overpotentials, as shown in Figure 4(d2). The relation between low DA concentrations and the SWV responses of the Au ME and the ACNE/Au ME were linearly fitted, as shown in Figure 4(d3). The slope of the fitted curve for the Au ME was 0.00094 μA/μM, and the slope of the fitted curve for the ACNE/Au ME was 0.0018 μA/μM, which demonstrated that the ACNE/Au ME could also detect DA solutions in the lower concentration range with high sensitivity and good stability with the SWV detection method. In conclusion, ACNEs can improve the sensitivity and stability of Au MEs for the detection of DA solutions using the SWV method.

In addition to the DPV and SWV detection methods described above, we also investigated the effects of ACNE modification on the sensitivity of the Au microelectrode to detect DA using NPV and LSV. NPV is an electrochemical detection technique that applies a series of linearly increasing pulses at a constant potential, contributing to the background current during measurements in the same way as other pulse techniques. It has been shown that rectangular pulses can be used for more precise chemical differentiation and that NPV is finer for identifying voltage dependence [54]. LSV is similar to CV in that it can be used to quantitatively and qualitatively analyze the electrode reaction [55]. In addition, if the electrode reaction rate is slower than the polarization rate, the current peak does not appear. The Au ME showed no distinct peak profile when high concentrations of DA were detected by NPV, as shown in Figure 4(e1), whereas the ACNE/Au ME exhibited a distinct oxidation peak profile, as shown in Figure 4(e2). The relations between the concentration and the NPV responses detected by the Au microelectrode and the ACNE/Au ME were both fitted, as shown in Figure 4(e3). The slopes of the linear equation were 0.009 μA/μM with R^2^ = 0.995 for the Au microelectrode and 0.067 μA/μM with R^2^ = 0.996 for the ACNE/Au ME. The results showed that the sensitivity of the assay with the ACNE/Au ME was 7.4 times higher than that with the Au ME in the detection of highly concentrated DA solutions. In the detection of low-concentration DA solutions by NPV, the peak NPV current curve of the Au ME is shown in Figure 4(f1), and the peak current curve of the ACNE/Au ME for the detection of low-concentration DA solution is shown in Figure 4(f2). The relation between low-concentration DA solution and NPV responses is shown in Figure 4(f3). The detection slopes were 0.022 μA/μM for the Au ME and 0.18 μA/μM for the ACNE/Au ME. With the NPV detection method, compared to the Au ME, the detection sensitivity of the ACNE/Au ME was increased by 8-fold. Figure 4(g1,g2) show the effect of ACNE modification on the detection performance of the Au ME for high concentrations of DA by LSV, and the results show that the LSV curves swept for the ACNE/Au ME had more obvious current peaks, which indicates that the ACNE/Au ME had a faster response rate. The peak currents of the relations between concentration and the LSV responses of the Au microelectrode and the ACNE/Au ME were fitted, as shown in Figure 4(g3). The slope of the fitted curve for the Au microelectrode was 0.0038 μA/μM with R^2^ = 0.998, while the slope of the fitted curve for the ACNE/Au ME was 0.014 μA/μM with R^2^ = 0.986. The results showed that the sensitivity of the ACNE/Au ME was 3.7 times higher than that of the Au ME in the detection of highly concentrated DA solutions. Figure 4(h1) shows the LSV peak current curve of Au MEs in low-concentration DA solution, and Figure 4(h2) shows the LSV peak current curve of the ACNE/Au ME in low-concentration DA solution. In the relation between concentration and LSV responses shown in Figure 4(h3), the slope of the fitted curve for the Au microelectrode was 0.0045 μA/μM, while the slope of the fitted curve for the ACNE/Au ME was 0.09 μA/μM. These results showed that the detection sensitivity of the ACNE/Au ME was 20 times higher than that of the Au ME in the detection of low-concentration DA solution. In conclusion, after the exploration of NPV and LSV electrochemical detection methods, the results show that the modification of ACNEs significantly improves the electrochemical performance of Au MEs, including the sensitivity and response speed of detecting DA.

**Figure 5:**
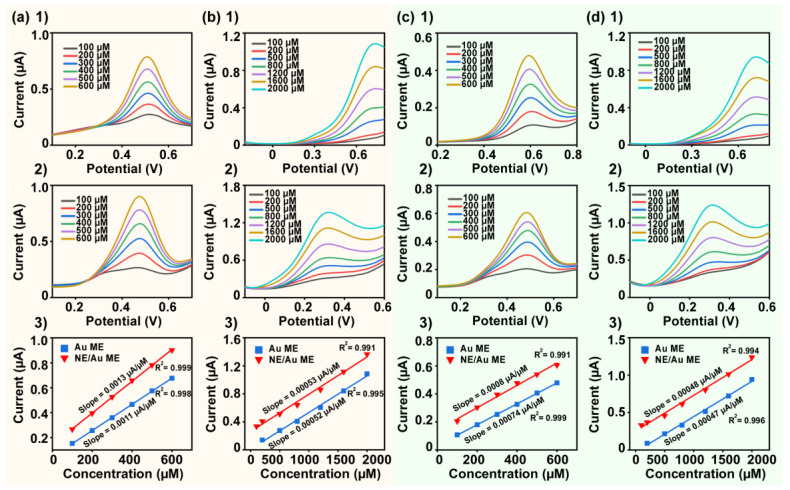
Characterization of electrochemical properties of ACNE/Au ME in UA solution (100–600 μM) and AA solution (100–2000 μM). (**a1**) DPV curves of Au ME in UA solution; (**a2**) DPV curves of ACNE/Au ME in UA solution; (**a3**) peak currents of the relations between concentration and DPV responses of Au ME and ACNE/Au ME in UA solution. (**b1**) DPV curves of Au ME in AA solution; (**b2**) DPV curves of ACNE/Au ME in AA solution; (**b3**) peak currents of the relations between concentration and DPV responses of Au ME and ACNE/Au ME in AA solution. (**c1**) SWV curves of Au ME in UA solution; (**c2**) SWV curves of ACNE/Au ME in UA solution; (**c3**) peak currents of the relations between concentration and SWV responses of Au ME and ACNE/Au ME in UA solution. (**d1**) SWV curves of Au ME in AA solution; (**d2**) SWV curves of ACNE/Au ME in AA solution; (**d3**) peak currents of the relations between concentration and SWV responses of Au ME and ACNE/Au ME in AA solution.

We investigated the effect of the ACNE/Au ME on the electrochemical detection performance of UA and AA by DPV and SWV. Figure 5(a1) shows the peak DPV current curves swept by the Au ME in UA solutions in the concentration range of 100–600 μM. Figure 5(a2) shows DPV peak current curves swept by the ACNE/Au ME. By comparing the two DPV peak current curves, the results showed that the ACNE/Au ME detected a lower overpotential of UA. To compare the sensitivity of the two electrodes for the detection of UA using the DPV method, we fitted a curve of the relation between UA and DPV responses, as shown in Figure 5(a3). The slope of the fitted curve for the Au ME was 0.0011 μA/μM, R^2^ = 0.998, and the slope of the fitted curve for the ACNE/Au ME was 0.0013 μA/μM, R^2^ = 0.999. The results showed that the ACNE/Au ME exhibited lower overpotentials and closer sensitivity than the Au ME. In the AA solution in the concentration range of 100–2000 μM, Figure 5(b1) shows the DPV peak current curve tested with the Au ME, and Figure 5(b2) shows the DPV peak current curve tested with the ACNE/Au ME. We found that the reaction overpotential window for AA detection by the Au ME was between 0.6. and 0.7 V. The larger detection voltage in this interval was prone to produce side reactions and interfere with the experimental results. The ACNE/Au ME greatly reduced the overpotential of AA to between 0.2 and 0.4 V. The reduction in overpotential is conducive to improving the detection accuracy of the electrode. In addition, we obtained slopes of 0.00052 μA/μM, R^2^ = 0.995, for the Au ME and 0.00053 μA/μM, R^2^ = 0.991, for the ACNE/Au ME by linearly fitting the relations between concentrations and the DPV responses of the two electrodes, as shown in Figure 5(b3). The results indicated that the ACNE/Au ME exhibited a lower overpotential and more consistent sensitivity than the Au ME when utilizing the DPV method for AA detection. The above results illustrate that ACNEs do not significantly enhance the sensing sensitivity of Au MEs in the detection of UA or AA concentrations using the DPV method, but they effectively reduce the detection overpotential. Overall, the modification of ACNEs facilitated the detection of UA and AA by the DPV method in Au MEs.

In addition, we investigated the electrochemical detection performance of the ACNE/Au ME for UA and AA using the SWV method. Figure 5(c1) shows the SWV characteristic curves of the Au ME in ΜA solutions in the concentration range of 100–600 μM, and Figure 5(c2) shows the SWV characteristic curves of the ACNE/Au ME in UA solutions in the concentration range of 100–600 μM. Similarly to DPV, the modification of ACNEs resulted in a lower overpotential for UA detection by SWV. In the relations between concentration and SWV responses shown in Figure 5(c3), the slope for the Au ME was 0.00074 μA/μM, R^2^ = 0.999, and the slope for the ACNE/Au ME was 0.0008 μA/μM, R^2^ = 0.991. Similarly, Figure 5(d4) shows the SWV characteristic curves of the Au ME in AA solutions in the concentration range of 100–2000 μM, and Figure 5(d2) shows the SWV characteristic curves of the ACNE/Au ME in AA solutions in the concentration range of 100–2000 μM. The results showed that the modification of ACNEs resulted in a significant reduction in reaction overvoltage in the detection of AA by the SWV method. In the fitted linear equations (Figure 5(d3)), the slope of the Au ME was 0.00047 μA/μM, R^2^ = 0.996, and the slope of the ACNE/Au ME was 0.00048 μA/μM, R^2^ = 0.994. In summary, the ACNE-modified Au ME improved the SWV and DPV sensing performance of UA and AA, which was mainly manifested by the larger reduction in overpotential.

**Figure 6:**
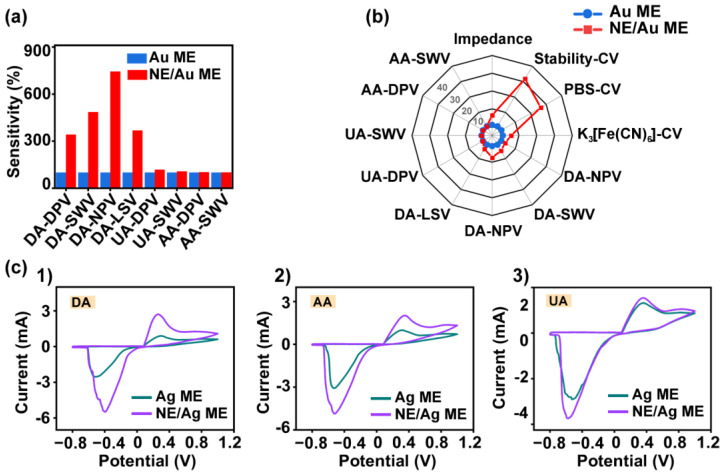
(**a**) Comparative histograms of the sensitivity of Au microelectrode and ACNE/Au ME for testing DA, AA, and UA by four electrochemical methods, DPV, SWV, NPV, and LSV, normalized by the results of the Au ME test as 100%. (**b**) Radar plots showing comparative plots of multiple properties of Au ME and ACNE/Au ME, including impedance, stability, oxidation peaks at overpotentials in PBS and K_3_[Fe(CN)]_6_ solution, and the sensitivity of the four electrochemical methods of detection, DPV, SWV, NPV, and LSV. (**c**) Comparison of the performance of Ag ME with and without ACNE modification for the detection of DA, AA, and UA by the CV method. (**c1**) CV curves of Ag microelectrode and ACNE/Ag ME in 100 μM DA solution. (**c2**) CV curves of Ag microelectrode and ACNE/Ag ME in 1000 μM AA solution. (**c3**) CV curve of Ag microelectrode and ACNE/Ag ME in 600 μM UA solution.

To compare the above experimental results more intuitively, we summarized the sensitivities of the three substances, DA, AA, and UA, detected by the four electrochemical methods into bar graphs, as shown in Figure 6a. The detection sensitivity of the ACNE/Au ME was normalized using the detection sensitivity of the Au ME as the 100% standard. Table 1 summarizes the sensitivity results for all electrochemical tests. In the experiments for detecting DA, the sensitivities of the Au ME modified with ACNEs were all greatly improved. Among them, the highest sensitivity enhancement of 7-fold was observed in the detection of DA using NPV. In addition, the Au ME modified with ACNEs showed a significant reduction in the detection of overpotentials when detecting both UA and AA, although it was close to that of the Au ME in terms of detection sensitivity. Further, Figure 6b summarizes the 12 properties of the Au ME and the ACNE/Au ME via radar plots to more visually compare the enhancement of the electrochemical properties of the Au ME after modification with ACNEs, and Table 2 complements the analysis of the inherent characteristics of the electrodes. Overall, the modification of ACNEs showed large enhancements of multiple electrochemical properties of the Au ME, especially for the improvement of its sensitivity to DA detection at low concentrations and the reduction in the overpotentials of DA, UA, and AA detection.

On the other hand, to illustrate the enhancing effect of ACNEs on the electrochemical properties of Ag-based materials, we supplemented the solution tests of the Ag ME and the ACNE/Ag ME. The Ag ME and ACNE/Ag ME were subjected to CV tests in different concentrations of DA, AA, and UA solutions. Comparing the CV curves of the Ag ME and the ACNE/Ag ME in 100 μM DA solution, the peak current of the electrode modified with ACNEs was significantly increased, as shown in Figure 6(c1). The CV curves comparing the Ag MEs and the ACNE/Ag MEs in 1000 μM of AA solution, as shown in Figure 6(c2), and in 600 μM of UA solution, as shown in Figure 6(c3), were similar. The ACNE-modified Ag MEs all had higher peak currents than the Ag MEs. This result suggests that ACNEs have the property of increasing the sensitivity of Ag MEs for the detection of DA, AA, and UA. In conclusion, in electrochemical sensing, metal electrodes modified with ACNEs have higher peak currents on all electrochemical characteristic curves than unmodified metal electrodes. In addition, the sharper peak curves and lower overpotentials indicate that ACNEs enhance the electrochemical oxidation reaction of the electrodes with good electrocatalytic activities for DA, AA, and UA, with a more significant enhancement of electrocatalytic activity and sensitivity for DA.

## 4. Conclusions

In this study, we proposed a synthetic ACNE and verified that, after ACNE modification, the low-frequency impedance of the metal electrodes was reduced and the electrochemical detection performance of the electrodes for DA, AA, and UA was improved. For these, the electrochemical detection methods included DPV, SWV, NPV, and LSV. In this work, ACNE nanoclusters were successfully modified on the surfaces of Ag and Au electrodes by the atomic layer deposition technique. Through electrochemical impedance analysis, it was verified that the modification of ACNEs reduced the low-frequency impedance of the metal electrodes. Compared with Ag-based microelectrodes, Au-based microelectrodes have better stability. In addition, by comparing the detection results of Au MEs and ACNE/Au MEs for different concentrations of DA in four electrochemical methods, including DPV, SWV, NPV, and LSV, it was verified that ACNEs had the effect of lowering the overpotential of the electrodes and improving their sensitivity in all four detection methods. Further, the detection performance of ACNEs on UA and AA was tested by DPV and SWV, and the results illustrated that the modification of ACNEs reduced the sensing overpotential of the Au ME and improved the catalytic activity of the electrode. Comparing the results of different electrochemical methods for the detection of DA, AA, and UA, the ACNE/Au ME showed good catalytic activity and high sensitivity and was able to produce faster and more significant responses to the analytes at lower overpotentials, with the detection of DA being more prominent. The ACNE/Au ME was capable of sensitively detecting sub-micromolar concentrations of DA solution. In summary, the proposed Au ME with ACNE coating in this study can enhance the electrochemical sensing performance of metal electrodes. This suggests the possibility of nanoenzyme coatings to enhance the electrochemical performance of microelectrodes, which is beneficial to develop advanced implantable biosensors for biomedical applications.

## Figures and Tables

**Table 1:** Electrochemical sensitivity analysis of Au ME and ACNE/Au ME.

Types of Electrochemical Detection	Sensitivity of Au ME (nA/μM)	Sensitivity of ACNE/Au ME (nA/μM)	Performance Multiplier (Set Au ME as 1)
DA-DPV	1.2	4.1	3.42
DA-SWV	0.74	3.6	4.86
DA-NPV	9	67	7.44
DA-LSV	3.8	14	3.68
UA-DPV	1.1	1.3	1.18
UA-SWV	0.74	0.8	1.07
AA-DPV	0.52	0.53	1.02
AA-SWV	0.47	0.48	1.01

**Table 2:** Intrinsic electrochemical characteristics of Au ME and ACNE/Au ME.

Intrinsic Characteristic	Impedance	Stability-CV (Peak Variance)	PBS-CV (Peak Current)	K_3_[Fe(CN)_6_]-CV (Peak Current)
Item				
Au ME	1,885,000 ohm	2.40 × 10^−8^ μA^2^	0.01089 μA	0.369 μA
ACNE/Au ME	295,500 ohm	7.55 × 10^−10^ μA^2^	0.2883 μA	2.07 μA
Performance multiplier (set Au ME as 1)	6.38	31.83	26.47	5.61

## Data Availability

Data are contained within the article.
